# Post-Kala-Azar Dermal Leishmaniasis as a Reservoir for Visceral Leishmaniasis Transmission

**DOI:** 10.1016/j.pt.2019.06.007

**Published:** 2019-08

**Authors:** Epke A. Le Rutte, Eduard E. Zijlstra, Sake J. de Vlas

**Affiliations:** 1 Department of Public Health, Erasmus MC, University Medical Center Rotterdam, P.O. Box 2040, 3000 CA Rotterdam, The Netherlands; 2 Department of Epidemiology and Public Health, Swiss Tropical and Public Health Institute, Socinstrasse 57, Basel 4002, Switzerland; 3 University of Basel, Basel 4003, Switzerland; 4 Rotterdam Centre for Tropical Medicine, Bovenstraat 21, 3077 BB Rotterdam, The Netherlands

**Keywords:** post-kala-azar dermal leishmaniasis (PKDL), visceral leishmaniasis, elimination, transmission dynamics, xenodiagnosis

## Abstract

Post-kala-azar dermal leishmaniasis (PKDL) is a parasitic skin infection which can occur after visceral leishmaniasis (VL). Recent xenodiagnosis studies (Mondal *et al*., *Clin. Infect. Dis*., 2018) have uncovered the infectiousness of PKDL. When including this in a transmission model, PKDL cases appear as an important reservoir of infection, likely frustrating the VL elimination efforts on the Indian subcontinent.

Kala-azar, commonly known as visceral leishmaniasis (VL), is a neglected tropical disease that has been targeted on the Indian subcontinent (ISC) for elimination as a public health problem by 2020, that is, a reduction of incidence to <1 VL case per 10 000 of the population per year at (sub)district level. *Leishmania donovani* protozoa are transmitted by female sand flies and infect humans, who mostly remain asymptomatic. However, a small percentage (1–10%) develop symptomatic VL, which is fatal if left untreated [1]. After recovering from VL, about 2.5–20% of patients develop post-kala-azar dermal leishmaniasis (PKDL), a nonlethal skin condition that can last for years and appears in two main forms: nodular or macular [2]. The interventions to achieve the 2020 elimination target focus on timely diagnosis and treatment of VL cases, and vector control through indoor residual spraying of insecticide.

A crucial knowledge gap in the transmission dynamics of VL is the reservoir of infection. It is largely unknown if, and to what extent, asymptomatic and PKDL cases contribute to transmission, as has long been highlighted by the PKDL Consortium as one of the key topics for future research [3]. Infectious PKDL cases could be a long-lasting source of transmission and thereby frustrate elimination efforts. We have recently explored the significance of these unknowns using two mathematical transmission model variants: one with the main reservoir of infection in symptomatic cases, that is, both VL and PKDL (Model E0), and one with the main reservoir of infection in asymptomatic individuals (Model E1) [4]. Both models assume that 2.5% of treated VL cases develop PKDL (conservative assumption based on data from India [5]), and PKDL was assumed to be half as infectious as VL [6].

Mondal *et al*. (October 2018) have reported their findings of the long awaited xenodiagnosis studies, that is, exposing sand flies to individuals with different stages of disease and infection [7]. They found that, for 15 VL and 47 PKDL patients (comprised of 21 patients with nodular and 26 patients with macular PKDL), nodular PKDL was more likely (86%) and macular PKDL less likely (35%) to result in infected sand flies compared with VL (67%) [7]. When taking into account the distribution of the different forms of PKDL in this region, with 48% being macular and 52% nodular [8], the relative infectiousness of PKDL is 0.9 compared with VL (1.0), making PKDL cases nearly equally infectious.

Figure 1 shows the implications of these new findings in a typical high-endemic setting (precontrol incidence of 10 VL cases/10 000/year), using both model variants now incorporating the empirically based PKDL infectiousness of 0.9 relative to that of VL. Clearly, 5 years of intensive WHO strategy (solid curves) led to a rapid incidence reduction to about the target of 1/10 000/year, but when the WHO strategy changed to less-intense interventions after year 5, incidence reductions slowed down. The relative contribution to transmission of the different infection stages during the WHO strategy (two panels above) indicate that the contribution of PKDL cases to transmission more than triples for both models after 5 years of intensive WHO interventions, clearly highlighting the need for a PKDL control strategy. When adding a hypothetical PKDL control strategy (here: preventing 95% of PKDL) to the existing WHO strategy, the elimination target is achieved as much as 8 years earlier for model E0 (dashed line). Elimination of transmission (0/10 000/year) will also be achieved several years sooner. The relative contribution of PKDL to transmission, as well as the impact of a PKDL control strategy, will be larger in settings where 20% instead of 2.5% of VL cases develop PKDL.

**Figure 1:**
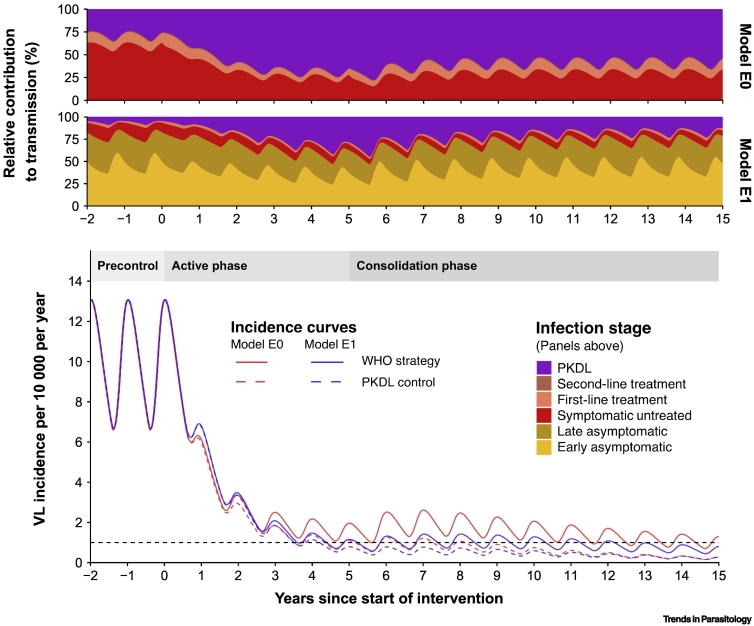
Visceral Leishmaniasis (VL) Incidence Curves during Interventions and the Corresponding Change in Relative Contribution to Transmission of Different Infection and Disease Stages during the WHO Strategy. The precontrol VL incidence (year –2–0) in a high-endemic setting (10/10 000/year) is followed by 5 years of interventions in the WHO active phase (years 0–5) and then 10 years of interventions in the WHO consolidation phase (years 5–15). The dashed lines represent the incidence curves for which the regular WHO strategy is combined with the post-kala-azar dermal leishmaniasis (PKDL) control strategy (years 0–15). The black horizontal dashed line depicts the 1/10 000/year target of elimination as a public health problem. In Model E0 only symptomatic individuals with VL and PKDL contribute to transmission; in Model E1, asymptomatic individuals also contribute to transmission. The early asymptomatic stage represents individuals that are PCR-positive and negative in the direct agglutination test (DAT); the late asymptomatic stage represents individuals that are PCR-positive and DAT-positive (as fitted to the KalaNet dataset, India [9]). Late-asymptomatic individuals are considered to be twice as infectious compared with early-asymptomatic individuals [10]. A small percentage of late-asymptomatic individuals become symptomatic, after which most receive first-line treatment. In case of treatment failure, a second-line treatment is administered. Individuals in the treatment stages are considered half as infectious compared with those in the symptomatic untreated stage [6]. Further information about the models is presented in [4].

PKDL control is currently not included in the main intervention programs, and the options are limited. Straightforward but operationally difficult strategies could entail active PKDL case-finding and treatment, long-term follow-up of VL patients, patient education on the need to report to the clinic in case of a rash, and educating community workers on recognizing PKDL, all requiring the availability of safe and effective treatment. For the future, we hope for better VL treatment, including a vaccine or immunomodulator to avert any PKDL development.

Obviously, we also eagerly await the outcomes of ongoing xenodiagnosis studies on the infectiousness of asymptomatic individuals to further complete the VL puzzle (helping us to choose between both model variants). If the model-estimated infectiousness of asymptomatic cases in Model E1 is true (i.e., 1–2% relative to VL), then several hundreds of asymptomatic patients need to be tested in order to identify the few that lead to infection of sand flies.
